# Supplementary material for: Bacterial Community Characteristics Shaped by Artificial Environmental PM2.5 Control in Intensive Broiler Houses
Source: Int J Environ Res Public Health. 2022 Dec 30;20(1):723. doi: 10.3390/ijerph20010723 (PMC9819255; doi:10.3390/ijerph20010723)
Supplement: Supplementary file 1 [file ijerph-20-00723-s001.zip › ijerph-2103497-supplementary.pdf]

Supplementary Materials:

Figure S1: The relative abundance of airborne bacteria at the phylum level inside and outside the house.

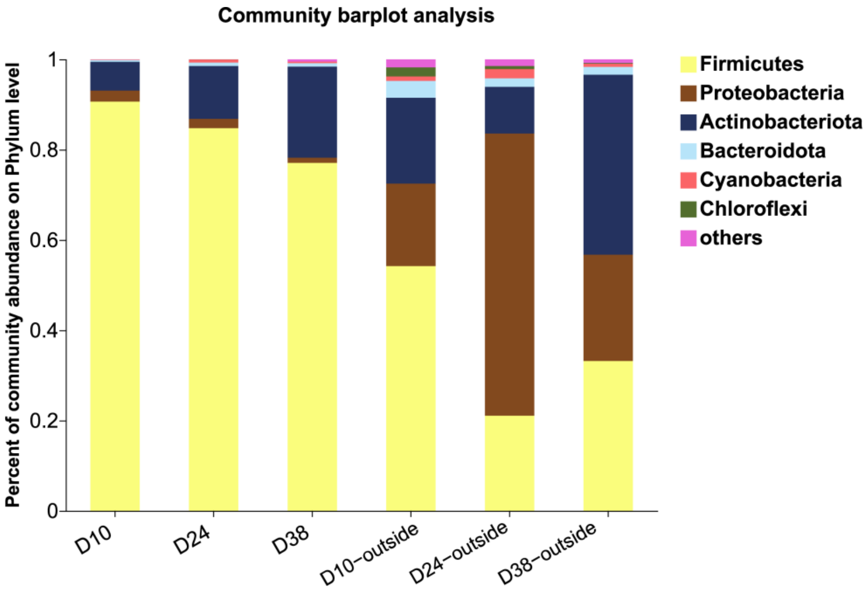

Figure S2: The ternary phase diagram of indoor bacteria.

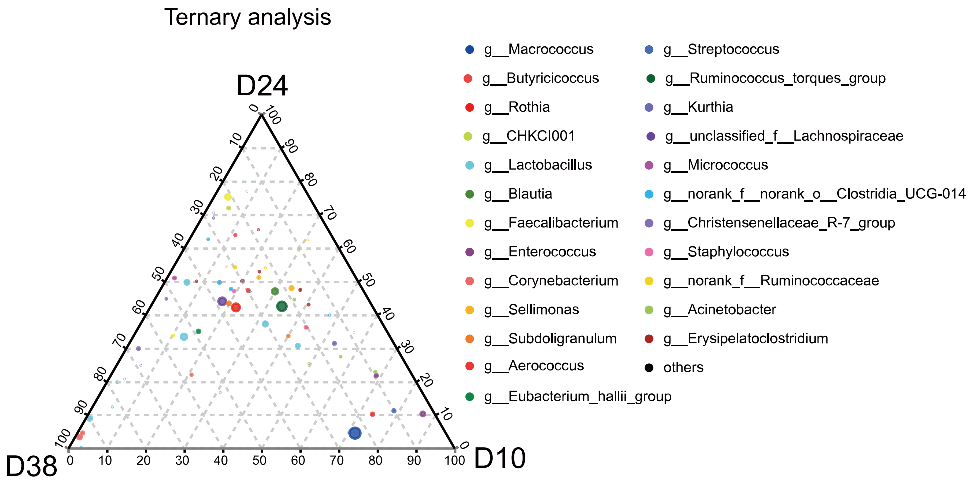

Figure S3: LEfSe hierarchical tree diagram of multi-level species of indoor bacteria.

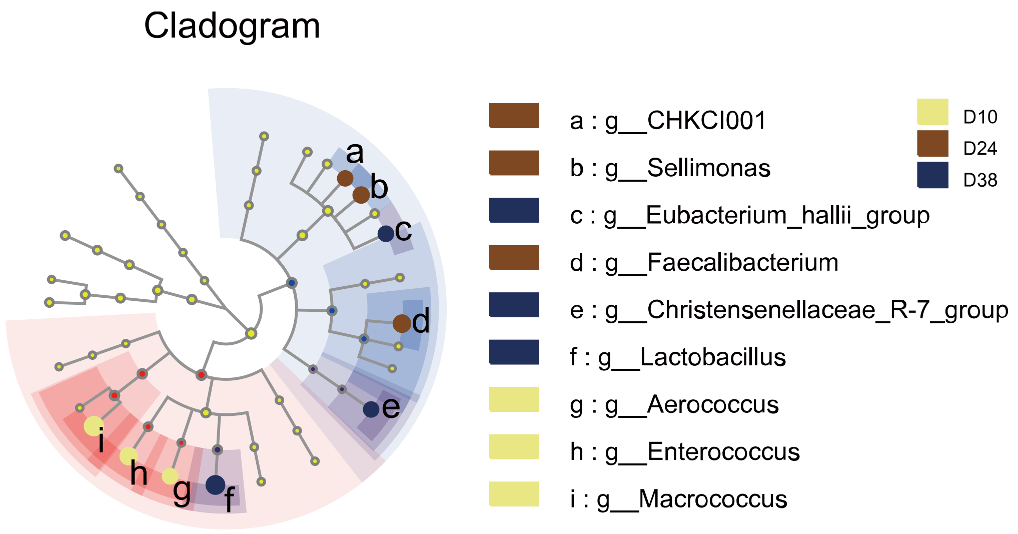

Figure S4: The relative abundance of manurial and indoor airborne bacteria at the phylum level.

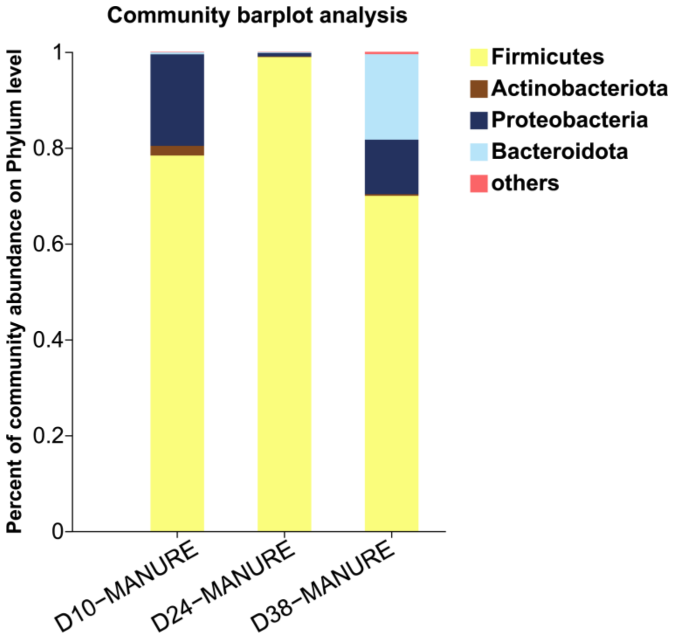

Table S1: Related data table of db-RDA.

	CAP1	CAP2	R^2	P_values
PM2.5	-0.6396	0.7687	0.9516	0.003
Temp	-0.9602	0.2793	0.9163	0.005
Hum	0.7915	-0.6112	0.9279	0.003
NH_3	-0.9321	0.3621	0.9220	0.007
CO_2	-0.9126	0.4088	0.9577	0.001
Wind	0.9353	-0.3537	0.8946	0.010
